# Optimal surgical approach for mid-transverse colon cancer: a systematic review and meta-analysis

**DOI:** 10.1007/s10147-024-02486-3

**Published:** 2024-04-29

**Authors:** Beshoy Effat Elkomos, Philopateer Effat Alkomos, Mina Fransawy Alkomos, Sameh Ahmed, Safa Owhida Baqar, Muhammad Faran Raza Bhatti, Rao Junaid, Muddasir Hassan, Muhammad Mazhar, Joseph Hanna, Guirgis Boushra Ebeidallah, Ayman Hossam eldin Abd el monaem Ali

**Affiliations:** 1 General Surgery Department, Ain Shams University Hospital, Cairo, Egypt; 2 Faculty of Medicine, Ain Shams University Hospital, Cairo, Egypt; 3 Gastroenterology Department, St. Joseph’s University, Paterson, NJ USA; 4 General and Emergency Surgery Department Northwick Park Hospital Healthcare NHS Trust, London North West University, London, UK; 5 Emergency Medicine Manchester Royal Infirmary, Manchester Foundation Trust, Manchester, UK; 6 Emergency Department Royal Derby Hospital, University Hospitals of Derby and Burton NHS Foundation Trust, Derby, UK

**Keywords:** Extended hemicolectomy, Transverse colectomy, Mid-transverse colon cancer

## Abstract

**Background and aim:**

The incidence of cancer colon has increased dramatically. In addition, the database lacks a review to analyze the outcomes of surgeries for mid-transverse colon cancer with several recent controversial studies. We aimed to compare the outcomes of extended hemicolectomy versus transverse colectomy for mid-transverse colon cancer.

**Method:**

PubMed, Scopes, Web of Science and Cochrane Library were searched for eligible studies from inception to 1 December 2022 and a systematic review and meta-analysis were done to detect.

**Results:**

According to eligibility criteria, 8 studies (2237 patients) were included in our study. The pooled results of the included studies showed no difference in the 5-year OS, 3-year DFS and 5-year DFS between the two types of surgery (5-year OS, RR = 1.15, 95% CI 0.94–1.39, *P* = 0.17), (3-year OS, RR = 0.96, 95% CI 0.88–1.06, *P* = 0.42) and (5-year DFS, RR = 1.21, 95% CI 0.91–1.62, *P* = 0.20). In addition to that, the recurrence rate and the incidence of complications were similar in the two groups (Recurrence rate, RR = 1.08, 95% CI 0.62–1.89, *P* = 0.79) and (Complications, RR = 1.07, 95% CI 0.74–1.54, *P* = 0.72). However, the number of LN harvest and the time of the operation were more in case of extended hemicolectomy.

**Conclusion:**

Despite harvesting less LN, transverse colectomy has similar oncological outcomes to extended hemicolectomy for mid-transverse colon cancer. In addition to that, there was no significant difference in the incidence of complications between the two surgeries.

## Introduction

In the past few years, the incidence of cancer colon has increased dramatically [1]. In addition to that, colon and rectal cancer altogether is considered the second leading cause of cancer-related mortality worldwide [2]. Transverse colon cancer, which accounts for 10% of all colorectal cancer [3], is defined as cancer that occurs in hepatic flexure, mid-transverse or splenic flexure. [4]

Several studies and reviews compared the outcomes of extended hemicolectomy and transverse colectomy in the management of transverse colon cancer as one unit [5, 6]. However, the database lacks a review to analyze the outcomes of surgeries for mid-transverse colon cancer with several recent controversial studies. Some of these studies reported equal long-term outcomes for extended colectomy and transverse colectomy for mid-transverse colon cancer [7–9]. Other studies reported that transverse colectomy is associated with a higher overall incidence of post-operative complications and a higher recurrence rate [10, 11]. Thus, it can be said that the optimal surgical procedure for mid-transverse colon cancer is not yet established.

Our aim was to detect the appropriate surgical intervention for mid-transverse colon cancer by comparing the outcomes of extended hemicolectomy and mid-transverse colectomy by performing a systematic review and a meta-analysis of the literature.

## Patients and methods

### Search strategy

PubMed, Web of Science and Scopus were searched for data from inception to 1 May 2022 with the following terms: Neoplasms, Transverse Colon and Colectomy. More searches by Google Scholar have been used to supplement the search with the sites mentioned above. All studies were reviewed by two authors (Elkomos, B. E. and Alkomos, P. E.) according to the inclusion criteria. Abstract-based eligibility studies were obtained, and the manuscripts were fully reviewed.

### Inclusion and exclusion criteria

The included studies should be (1) randomized controlled trials and prospective or retrospective cohort studies; (2) the target population were patients who were diagnosed with mid-transverse colon cancer; (3) studies designed to compare the outcome of extended colectomy versus transverse colectomy for mid-transverse colon cancer as a primary aim and (4) studies providing a sufficient data of the methods and baseline characteristics. The following types of studies were not included in our study: (1) reviews, case reports and case series; (2) unrelated or in vitro studies; (3) studies missing a comparison group.

### Outcomes of interest

Our main outcome was to detect patient outcomes (early post-operative mortality, overall survival, disease-free survival, recurrence rate and a number of lymph node harvests, for extended colectomy versus transverse colectomy for mid-transverse colon cancer. In addition to that, we compared operative details (time of the operation, operative blood loss and hospital stay) and post-operative complications (overall incidence of complications, leakage, ileus and surgical-site infection) for the two types of operation as a secondary outcome.

### Data extraction

We extracted data on study characteristics (author, year of publication, country of operation, study period and follow-up time), patient characteristics (age, sex and ASA stage), operative details (laparoscopic or open technique, elective or emergency surgery, time of the operation, blood loss during the operation and the hospital stay), characters of the tumor (tumor stage or histology, lymphatic, vascular perineural invasion) and the patient’s outcome (early post-operative mortality overall survival, disease-free survival, recurrence rate and the number of lymph node harvest). The data were extracted by 2 investigators (Elkomos, B. E. and Alkomos, P. E.) independently.

### Statistical analysis

The meta-analysis was performed according to Cochrane Handbook for Systematic Reviews of Interventions [12], which is recommended by the Cochrane Collaboration. For all the results included, the pooled risk ratios (RRs) and their corresponding 95% confidence intervals (CIs) were calculated with fixed effects models. However, if there was moderate or considerable heterogeneity (*I*^2^ > 40), random effects models were used to solve the heterogeneity between studies. All calculations for the current meta-analysis were performed with Review Manager 5.4 for Windows (Cochrane Collaboration, Oxford, UK).

## Results

### Characteristics and quality assessment of eligible studies

**Figure. 1:**
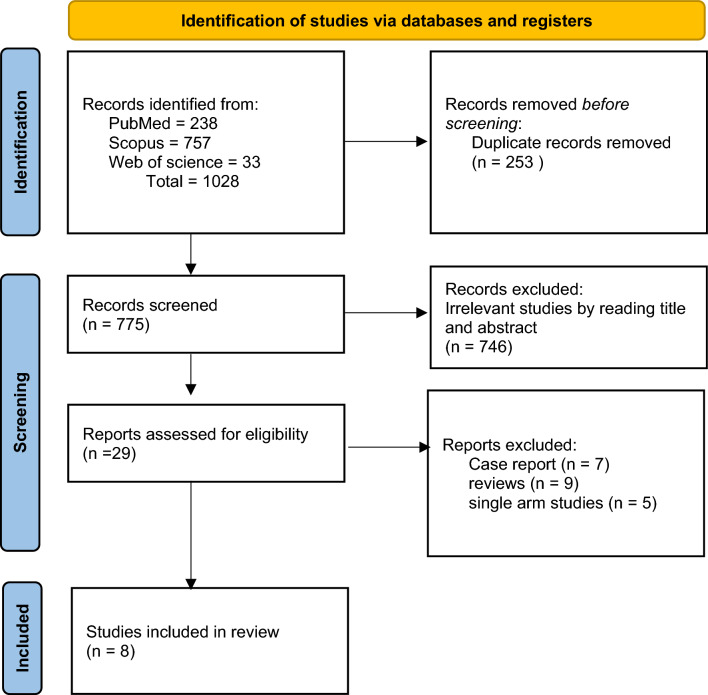
PRISMA flow diagram

As illustrated in the flow diagram (Fig. 1), 1028 articles were revealed using the following search string: Neoplasms” AND “Colon, Transverse’’ AND “Colectomy”. After careful selection, according to our eligibility criteria, 8 studies [7–11, 13–15] with 2237 participants were included in the meta-analysis. These trials included seven retrospective cohort studies and only one prospective study. Patients’ baseline data including [number, age, sex and ASA]. In addition to that, the approach of the surgery (laparoscopic or open, elective or emergency) and the characteristics of the tumor (tumor stage or histology, lymphatic, vascular perineural invasion) were comparable between the two groups in all studies (Table 1).

**Table. 1:** Basic data of the included studies

Author and publication year	Country	Study design	Study period	Follow up (months)	Arm	Sample size (*n*)	Age (year)	Gender: M/F(*n*)	Lap/open	Elective/emergency	ASA (I/II/III)(*n*)	AJCC TNM score, stage (I/II/III/IV)(*n*)	Histology (Adenocarcinoma/Mucous)	Vascular invasion (present/absent) (*n*)	Lymphatic invasion (present/absent) (*n*)	Perineural invasion (present/absent) (*n*)
Leijssen (2018) [7]	USA	Retrospective cohort	2004–2014	48.6	SC	38	75.4 (61.6–82.2)^b^	18//20	N/A	N/A	2.32 ± 0.56	10/21//7/0	34//2	8//30	13//25	4//34
					EC(Rt/Lt)	65	68.6 (57.7–81.1)^b^	35//30	N/A	N/A	2.44 ± 0.50	22/29//14/0	48//8	13/52	22//43	7//58
Matsuda (2018) [13]	Japan	Retrospective cohort	2007–2017	37	SC	34	73 (44–84)^b^	14//20	34//0	N/A	3/26//5/0	17/10//7/0	N/A	19/15	15/19	N/A
					EC(Rt/Lt)	38(38//0)	73 (41–93)^b^	22//16	38//0	N/A	4//29//5/0	16/12//10/0	N/A	23/15	17/21	N/A
Milone (2020) [10]	Italy	Retrospective cohort	2006–2016	3.6YS	SC	388	71.72 ± 12.88^a^	194/192	164/224	360/28	42/189/142/11	N/A	N/A	N/A	N/A	N/A
					EC(Rt/Lt)	1141	70.46 ± 11.03^a^	617/524	632/509	1049/92	129/594/394/27	N/A	N/A	N/A	N/A	N/A
Almoregy (2021) [11]	Egypt	prospective study	2015–2020	N/A	SC	40	55 (29–80)^b^	26/14	40/0	N/A	N/A	12/12//16	35/5	N/A	N/A	N/A
					EC(Rt/Lt)	80	55 (29–80)^b^	52/28	80/0	N/A	N/A	24/28//28	70/10	N/A	N/A	N/A
Chow (2021) [14]	China	Retrospective cohort	2000–2020	42.5	SC	23	75 (68–81)^b^	11//12	9//14	18/5	9/8/5/1	3/13/6/1	22/0	2//21	2//21	0//23
					EC(Rt/Lt)	84	70.5 (61–78)^b^	41//43	43//41	59/25	35/29/17/3	2/42/34/6	75/9	12//72	18//66	7//77
Park (2021) [8]	Korea	Retrospective cohort	2005–2015	N/A	SC	37	66.7 ± 10.9^a^	23/14	21/16	37/0	N/A	N/A	N/A	N/A	7//30	13//24
					EC(Rt/Lt)	70	69.0 ± 10.6^a^	36/34	56/14	70/0	N/A	N/A	N/A	N/A	10//60	19//51
Iguchi (2022) [15]	Japan	Retrospective cohort	2008–2019	N/A	SC	94	77 (30–92)^b^	42/54	94//0	94//0	N/A	47/24/23/0	N/A	N/A	N/A	N/A
					EC(Rt/Lt)	35(35/0)	73 (41–89)^b^	15/20	35/0	35//0	N/A	11/14/10/0	N/A	N/A	N/A	N/A
Sun (2022) [9]	China	Retrospective cohort	2012–2020	N/A	SC	40	58.5 ± 12.5^a^	15//25	40//0	N/A	4/24/12/0	3/19/17/0	33//7	N/A	N/A	3//37
					EC(Rt/Lt)	30(20/10)	61.9 ± 12.3^a^	17//13	30//0	N/A	5/15/10/0	2/18/10/0	29//1	N/A	N/A	3//27

^a^The results are presented as means and standard deviation

^b^The results are presented as median and range

### Primary outcome

#### Patient outcomes

##### Post-operative mortality

**Figure. 2:**
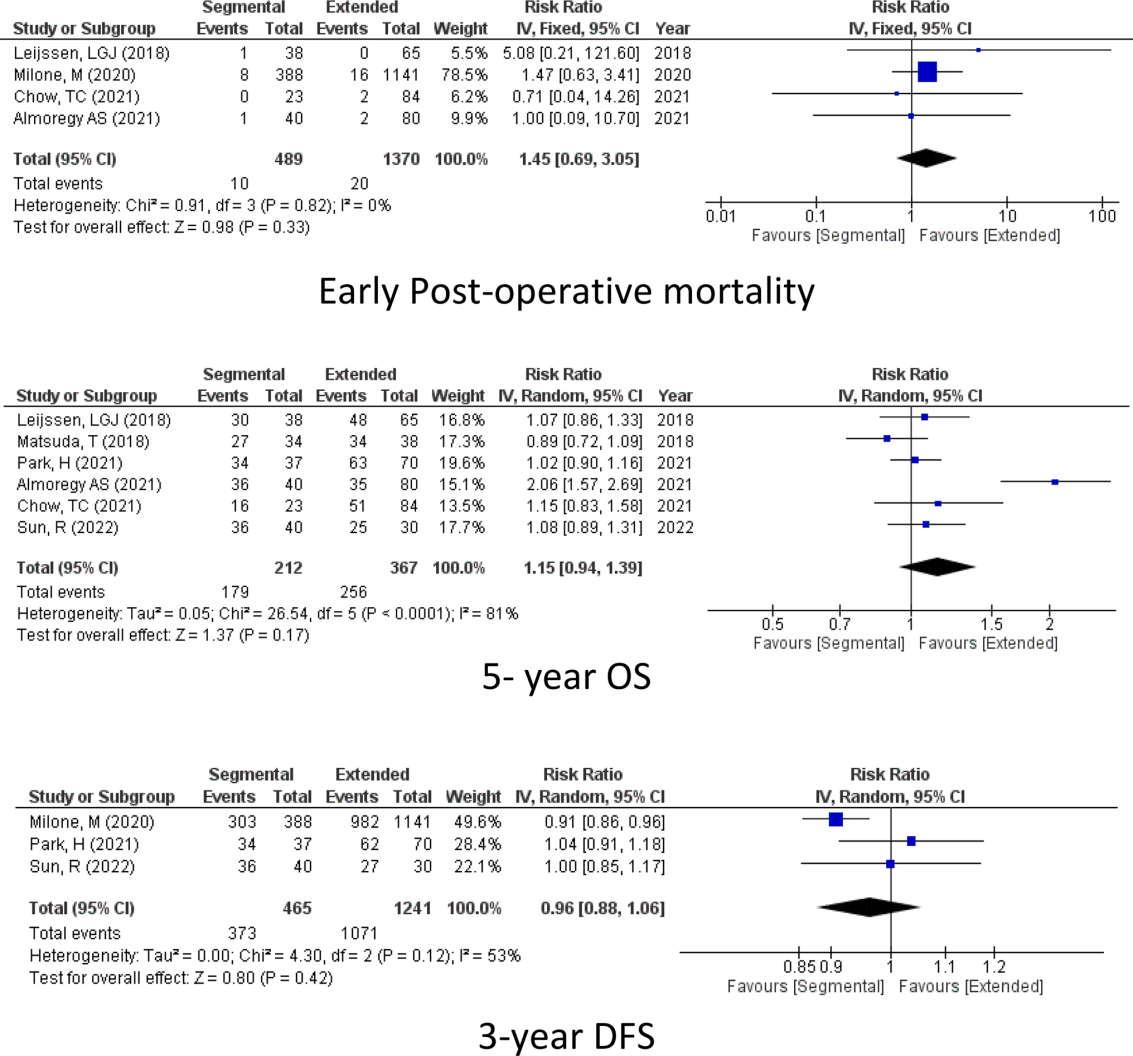

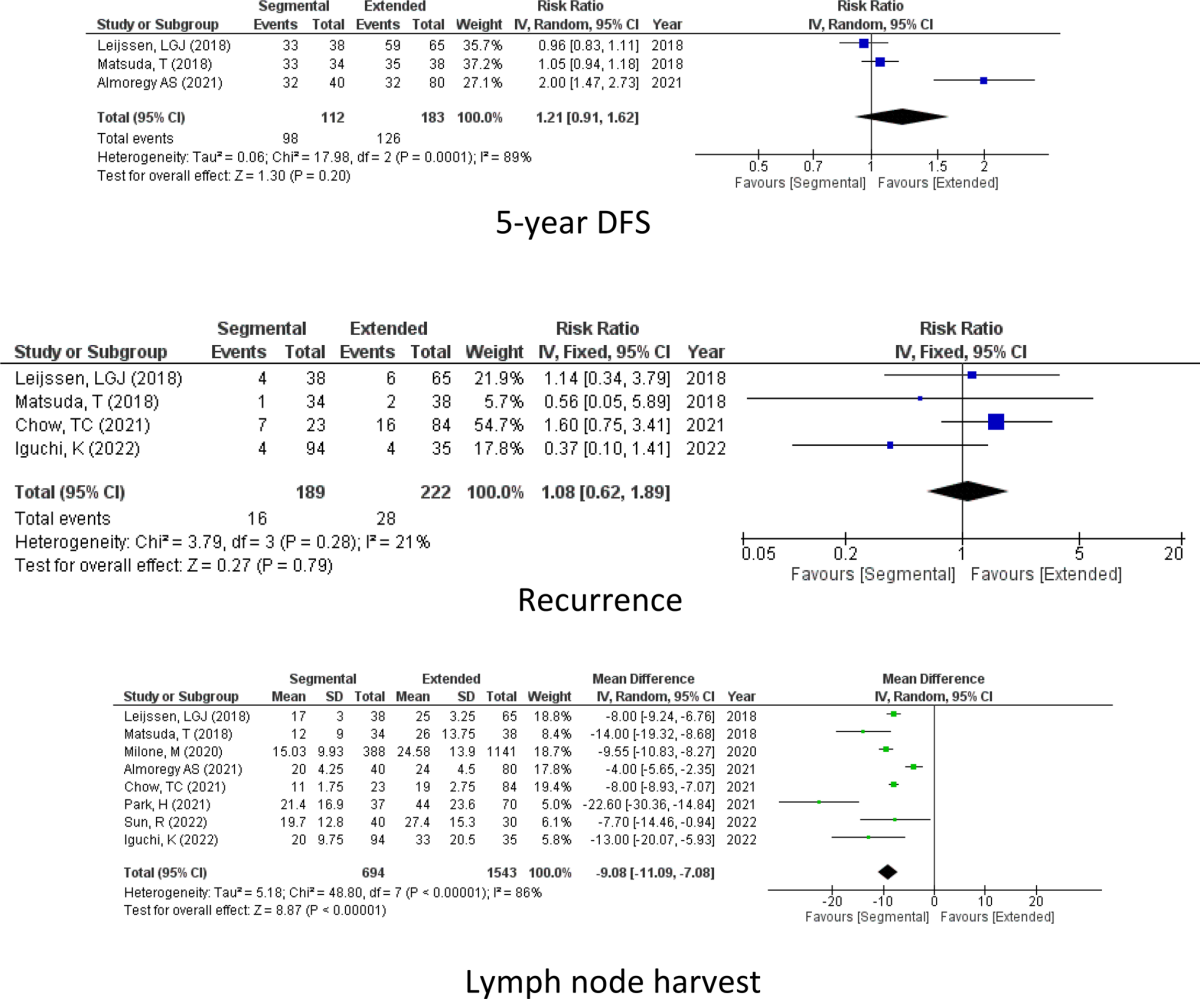
Oncological outcomes

According to 4 of the included studies (1859 patients), there was no significant difference in the rate of early post-operative mortality between the extended hemicolectomy and segmental colectomy for mid-transverse colon cancer (Mortality, RR = 1.45, 95% CI 0.69–3.05, *P* = 0.33; *I*^2^ = 0%) Fig 2.

#### Overall survival

Moreover, as reported by 6 studies (579 participants), there was no difference in the 5-year OS between the two types of surgery (5-year OS, RR = 1.15, 95% CI 0.94−1.39, *P* = 0.17; *I*^2^ = 81%) Fig 2.

#### Disease-free survival

In addition to that, the 3-year DFS was reported in 3 studies (1444 patients) and the 5-year DFS was reported in 3 studies (224 patients) and showed equal rated of DFS between the two surgeries. (3-year OS, RR = 0.96, 95% CI 0.88–1.06, *P* = 0.42; *I*^2^ = 53%) and (5-year DFS, RR = 1.21, 95% CI 0.91–1.62, *P* = 0.20; *I*^2^ = 89%) Fig 2.

#### Recurrence rate

Turning to the recurrence rate after colectomy as reported by 4 studies (411 participants), no difference could be detected in the two groups (Recurrence rate, RR = 1.08, 95% CI 0.62–1.89, *P* = 0.79 *I*^2^ = 21%) Fig 2.

#### Lymph node harvest

However, the number of lymph node harvests in the case of right hemicolectomy was much higher than the in segmental colectomy as reported by 8 studies (2237 participants) (LN, mean difference = − 9.08, 95% CI − 11.09 to − 7.08, *P* = < 0.00001; *I*^2^ = 86%) Fig 2.

### Secondary outcomes

#### Operative details

##### Time of the operation

**Figure. 3:**
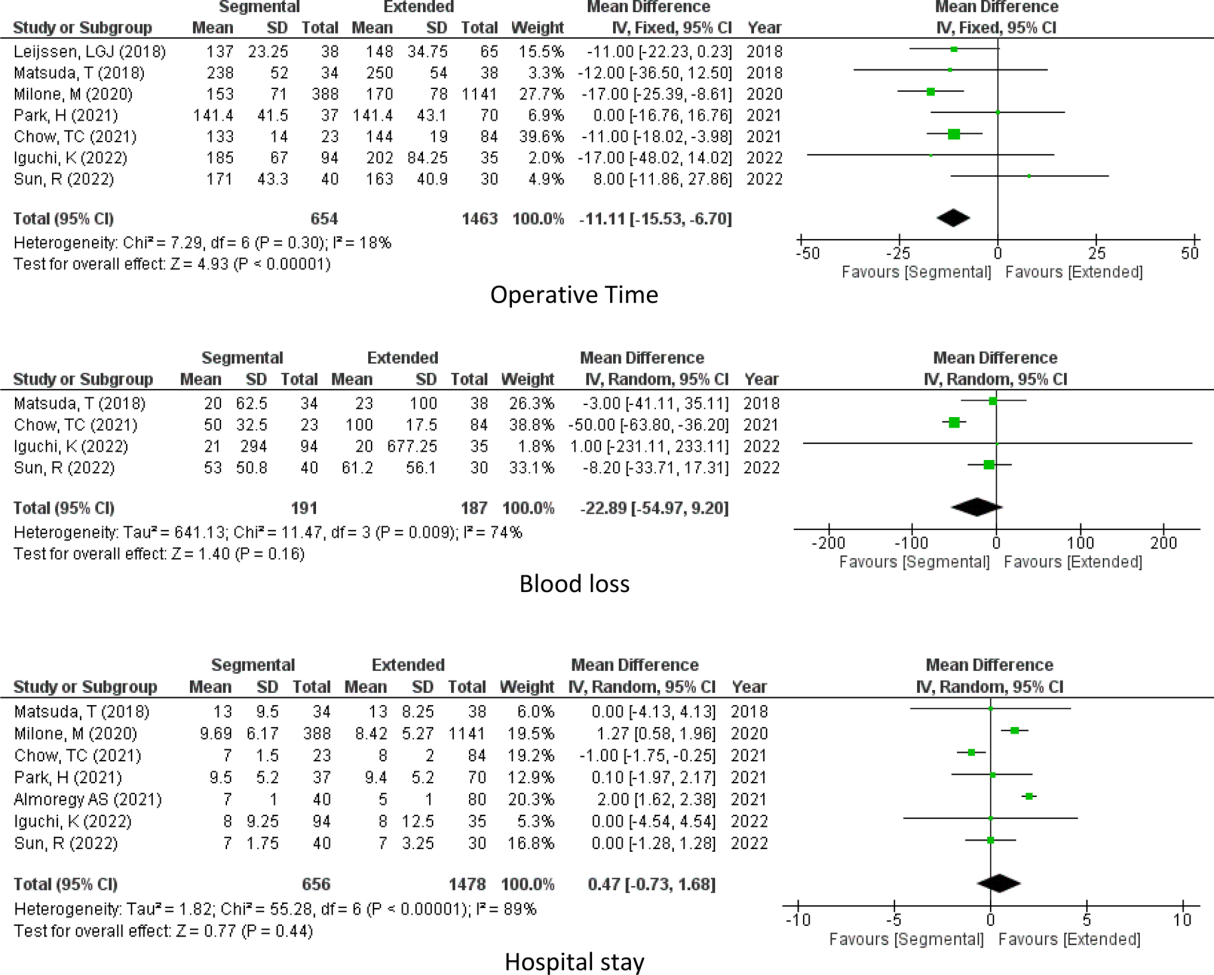
Operative details

Regarding the time of the operation as reported by 7 studies, it was longer in extended hemicolectomy than segmental hemicolectomy (operative time, mean difference = − 11.11, 95% CI − 15.53 to − 6.70, *P* < 0.00001; *I*^2^ = 18%) Fig 3.

#### Operative blood loss

Turning to the blood loss after the operation as reported by 4 studies (378 participants), no significant difference could be detected between the two operations (blood loss, mean difference = − 22.89, 95% CI − 54.97, 9.20, *P* = 0.16; *I*^2^ = 74%) Fig 3.

#### Hospital stay

According to 7 of the included studies, the length of hospital stay was similar in the two groups. (Hospital stay, mean difference = 0.47, 95% CI −0.73, 1.68, *P* = 0.44; *I*^2^ = 89%) Fig 3.

### Post-operative complications

#### Overall incidence of complications

**Figure. 4:**
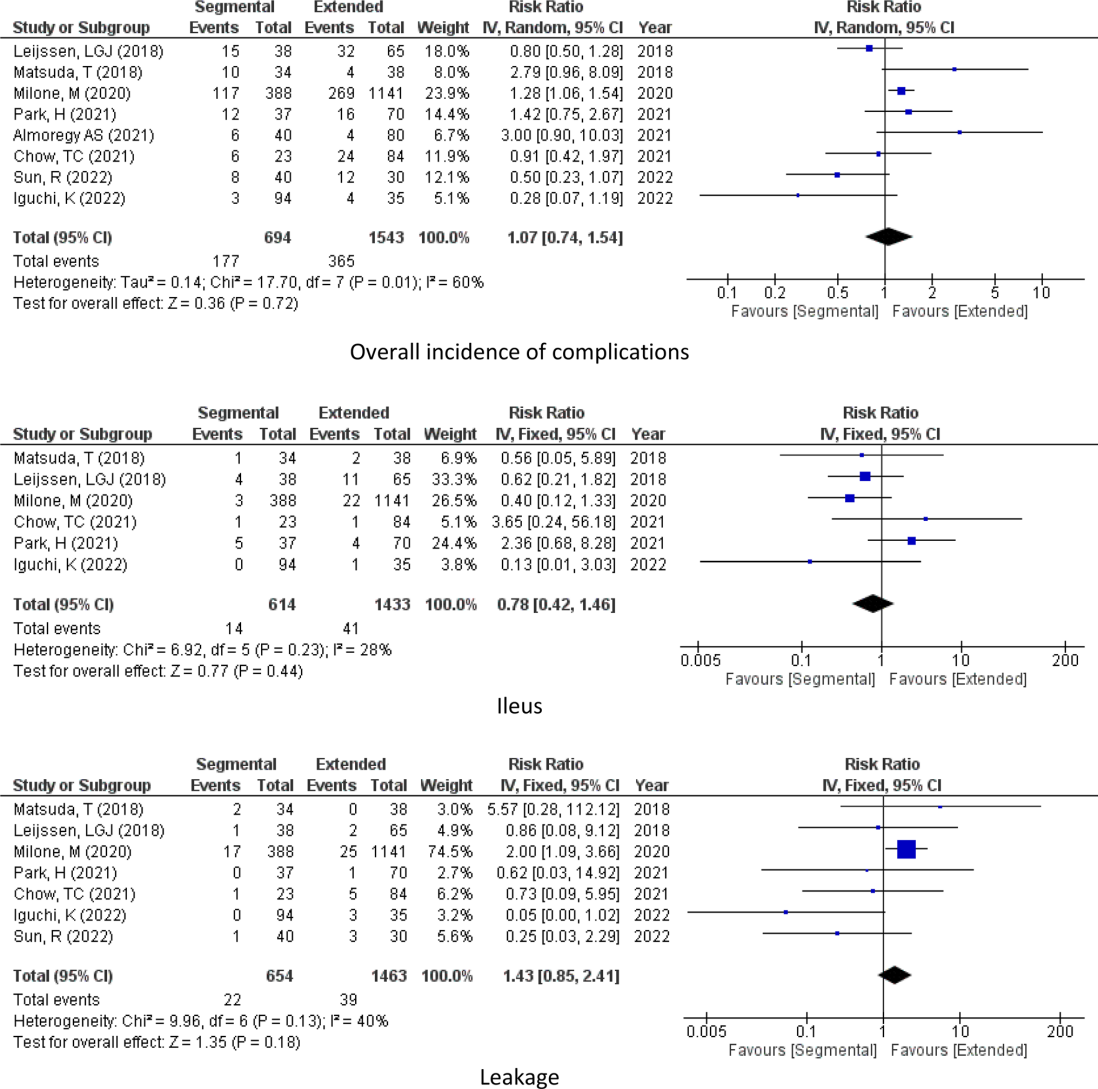
Complications

As reported by 8 studies (2237 patients), there was no significant difference in the incidence of complications between extended hemicolectomy and segmental colectomy (Complications, RR = 1.07, 95% CI 0.74–1.54, *P* = 0.72 *I*^2^ = 60%) Fig 4.

#### Leakage

Regarding post-operation leakage, according to the pooled results from 7 studies (2117 patients), it was similar for the two groups (Leakage, RR = 1.43, 95% CI 0.85–2.41, *P* = 0.18 *I*^2^ = 40%) Fig 4.

#### Ileus

In addition to that, post-operative ileus was similar in the surgeries as reported by 6 studies (2047 patients). (Ileus, RR = 0.78, 95% CI 0.42–1.46, *P* = 0.44 *I*^2^ = 28%) Fig 4.

#### Surgical site infection

Moreover, the incidence of complications was similar in the two groups as reported by 6 studies (1988 patients) (SSI, RR = 1.29, 95% CI 0.78–2.14, *P* = 0.32 *I*^2^ = 0%) Fig 4.

## Discussion

According to this meta-analysis, despite taking longer operative time and harvesting a larger number of lymph nodes, extended hemicolectomy is associated with similar oncological outcomes in comparison to transverse colectomy. In addition to that, no significant difference in the incidence of complications could be detected between the two types of surgeries. Extended right hemicolectomy is one the most common procedure for right colon diseases which includes ligature of three vessels (MCA, RCA and ilio-colic). Therefore, it is no surprise that the number of lymph nodes harvested in extended right hemicolectomy is more than that in transverse colectomy with a number ranging from of 24–33 LN and 12–20 LN respectively [7, 10, 13, 15]. However, as reported by Park et al. [8], metastasis to LNs along the right colic artery was about 10% of the patients with mid-transverse colon cancer, while there was no metastasis to LNs along the ileocolic artery in those patients. And according to Matsuda et al [13], no lymph node metastasis occurred around the right colic and ileocecal artery. Moreover, Milone et al. [10] and Almoregy et al [11] reported a similar number of positive LN in the two groups despite the larger number of LN harvests in the case of extended hemicolectomy. As reported by Chow et al. [14], the smaller number of LN is sufficient for proper staging According to our meta-analysis, overall survival, disease-free survival, and recurrence rate were similar in the two groups. Thus, it can be said that there is no difference between the two types of surgery from the oncological point of view. As reported by Leijssen et al [7] and Matsuda et al [13], there is no difference in the length of the two operations. However, in line with what was reported by other studies [10, 11, 15], our study showed that the length of the operation was longer in extended hemicolectomy in comparison to transverse colectomy. Turning to blood loss during operation, Leijssen et al [7], reported that the need for blood transfusion was higher in the extended group. However, the results were not statistically significant (18.4 vs. 29.2%, *P* = 0.223). In addition to that, according to other studies [13, 14], no statistically significant difference in the incidence of blood loss between the two groups and Milone et al [10] reported equal blood loss for the two types of surgeries. The pooled results of the included studies showed no difference in blood loss between extended and transverse colectomy. According to Milone et al [10] and Almoregy et al [11], there is a statistically significate decrease in the incidence of hospital stay for extended hemicolectomy. However, according to our study, there were no differences in hospital stays between the two groups. On the one hand, in line with what Almoregy et al [11] reported, Matsuda et al [13] reported a higher incidence of complications associated with transverse colectomy and this has been explained by the difference in surgeon’s preference or skills. Moreover, according to a study of 1529 patients [10], the overall incidence of complication was higher in the transverse group and the reason for that is transverse hemicolectomy needs the mobilization of both hepatic flexure and the splenic flexure which is technically challenging and may increase the risk of complications. However, Park et al [8] and Chow et al [14] reported no difference in the incidence of complications between those who underwent extended and transverse colectomy. On the other hand, Leijssen et al [7] and Iguchi et al [15] reported that patients who underwent extended colectomy group had a higher incidence of complications. However, the difference was statistically insignificant (49.1% vs. 39.5%, *P* = 0.337) and (11.4% vs. 3.2% *P* = 0.086) respectively. They explained that this observation might be due to a higher rate of post-operative ileus that is associated with extended hemicolectomy. According to the pooled results in our study, there was no difference in the incidence of complications between the two groups.

It is also worth mentioning that ileocecal junction resection surgery might affect bowel movement, enteric bacteria and nutrition state [13]. According to Su et al [16], in comparison to conventional extended right hemicolectomy, the incidence of diarrhea was lower in ileocecal junction-preserved surgery (*p* = 0.026). In addition to that, the defecation frequency was lower in this group on the 1st, 3rd, and 6th month post-operative (*p* < 0.05). However, according to the included studies, we cannot confirm the statistical superiority of ileocecal preservation for transverse colectomy in terms of bowel motions and diarrhea. To our knowledge, this is the first meta-analysis and systematic review to compare the outcomes of extended hemicolectomy versus segmental colectomy for mid-transverse colon cancer. However, we have to admit the presence of some limitations in this meta-analysis. First, all the included studies are cohort studies and there may be a sort of a surgeon’s selection bias that plays a role in the choice of the type of surgery for each individual case. Second, this study did not compare the outcomes of the laparoscopic versus open techniques for mid-transverse colon cancer. Lastly, we have to admit the presence of significant heterogeneity in some results. In conclusion, transverse colectomy has similar oncological outcomes to extended hemicolectomy for mid-transverse colon cancer. In addition to that, there was no significant difference in the incidence of complications between the two surgeries.

## Data Availability

The datasets used and analyzed during the current study are available from the corresponding author upon reasonable request.
